# Detention of HPV L1 Capsid Protein and hTERC Gene in Screening of Cervical Cancer

**Published:** 2013-06

**Authors:** Huang Bin, Wu Ruifang, Li Ruizhen, Liang Yiheng, Liu Zhihong, Li Juan, Wang Chun, Zhou Yanqiu, Weng Leiming

**Affiliations:** Department of Gynecology and Obstetrics, Shenzhen Hospital of Beijing University, Shenzhen 518036, China

**Keywords:** Cervical lesion, Human papilloma virus, Human telomerase RNA component, L1 capsid protein, Squamous cell carcinomas

## Abstract

***Objective(s):*** To investigate the expression of human papilloma virus (HPV) L1 capsid protein, and human telomerase RNA component (hTERC) in cervical cancer and the role of detection of both genes in screening of cervical cancer.

***Materials and Methods:*** A total of 309 patients were recruited and cervical exfoliated cells were collected. Immunocytochemistry was employed to detect HPV L1 capsid protein, and fluorescent in situ hybridization (FISH) was performed to detect the hTERC.

***Results:*** The expression of HPV L1 capsid protein reduced with the increase of the histological grade of cervical cells and was negatively related to the grade of cervical lesions. However, the expression of hTERC increased with the increase of the histological grade and positively associated with the grade of cervical lesions. The proportion of patients with L1(-)/hTERC(+) was higher in patients with histological grade of CIN2 or higher than that in those with histological grade of CIN1. The L1(+)/hTERC(-) and L1(-)/hTERC(-) were negatively related to the grade of cervical lesions. L1(-)/hTERC(+) was positively associated with the grade of cervical lesions. The L1/hTERC ratio increased. The negative predictive value of both HPV L1 and hTERC was higher than that of HPV L1 or hTERC, but there was no marked difference in the screening efficacy of cervical cancer among HPV L1, hTERC and HPV L1+hTERC.

***Conclusion:*** HPV L1 capsid protein and hTERC gene may serve as markers for the early diagnosis and prediction of cervical lesions. The increase in L1/hTERC ratio reflects the progression of cervical lesions to a certain extent.

## Introduction

Cervical cancer is the second leading common cause of malignancies in women (1-2). World Health Organization (WHO) introduced cervical cancer as the first cancer which is entirely caused by infection (3). The progression of cervical precancerous lesion into cervical cancer is mainly attributed to the sustained infection of human papilloma virus (HPV). However, not all the HPV infection of cervix develops into cervical cancer and the majority of HPV infection is subclinical. Thus, to identify HPV infection, patients with high risk of cervical cancer, and then to reduce over-medication are the challenges in clinical practice (4-8). Studies found that HPV L1 capsid protein and human telomerase RNA component (hTERC) gene were the biomarkers of prognosis of HPV infection, but detection of both HPV L1 capsid protein and hTERC gene is seldom reported as a strategy for the prediction of cervical cancer (9-14). In the present study, cervical exfoliated cells were collected from 309 patients and the expression of HPV L1 capsid protein and hTERC gene was determined. This study aimed at detecting the expression of HPV L1 capsid protein and hTERC gene in cervical exfoliated cells, and the role of detection of both HPV L1 capsid protein and hTERC gene in screening of cervical cancer.

## Materials and Methods

***Sample collection***

A total of 309 patients were recruited from the Shenzhen Hospital of Beijing University from January 2010 to May 2011, and cervical exfoliated cells were collected. The average age was 37.3±8.9 years (range: 18~67 years). Cytological examination was performed according to the Bethesda System (TBS) 2001. On the basis of cytological findings, for intraepithelial lesion or malignancy (NILM) was found negative in 72 patients, atypical squamous cells of undetermined significance (ASC-US) in 71, atypical squamous cells-cannot exclude high-grade squamous intraepithelial (ASC-H) in 19, Low grade squamous intraepithelial lesion (LSIL) in 80, high grade cervical squamous intraepithelial lesion (HSIL) in 49 and squamous-cell carcinoma (SCC) in 18. On the basis of pathological findings, cervical intraepithelial neoplasia (CIN) grade 1 was found in 168 patients, CIN grade 2/3 in 84, SCC in 24, normal cervical cells/cells of chronic cervicitis (absence of CIN or NILM) in 33. All patients had no conization of cervix, ureterectomy and a history of radiotherapy and/or chemotherapy. Patients with abnormal cells (≥ASC-US) or positive for HPV DNA also received vaginoscopy and biopsy. Diagnostic curettage was performed if necessary.

***Immunocytochemistry for HPV L1 Capsid Protein***

CytoReact cell/tissue HPV L1 detection kit (Myprice Medical Technology Co., Ltd, USA) was used to detect HPV L1 capsid protein according to manufacturer’s instructions. In brief, the smeared cells were fixed in 96% ethanol for 20 min and then dried. Subsequently, these cells underwent dehydration in 96%, 75% and 50% ethanol series and then in distilled water (2 min for each). Following washing with acid, pre-hybridization was done and inactivation of endogenous peroxidase was performed to remove the non-specific staining. Exogenous HPV L1 antibody was used to treat these cells followed by DNA hybridization and treatment with exogenous antibody again. Following hybridization, treatment with RNAase and peroxidase was performed. Staining was done with alkaline phosphatase and immunohistochemistry by AEC staining. Counterstaining and mounting were performed and sections were preserved at room temperature in dark. These sections were observed under a microscope. Sections with one nucleus stained red were regarded as positive for HPV L1. In addition, the negative and positive samples provided by the manufacture served as negative and positive controls, respectively and observed under a microscope. Sections with one nucleus stained red were regarded as positive for HPV L1.

***Detection of hTERC gene with fluorescent in situ hybridization (FISH)***

Probes (hTERC/CSP3 DNA) were provided by the Beijing Jinpujia Medical Technology Company. These probes can hybridize into 3q26.3, and red fluorescence (Tetramethylrhodamine) was presented. CSP3 served as a control probe, can hybridize into the centromere of chromosome 3 and presented green fluorescence. In brief, the cervical exfoliated cells were digested in collagenase B, treated with hypotonic solution and then fixed. Then cells were digested in pepsin. Following drying, cells were denaturated in formamide. The sections were hybridized with pre-denaturated probes overnight. After washing, the sections were observed under a fluorescence microscope. Normal cells had single nucleus and presented one red and one green signal. Cells with abnormal hTERC amplification had single nucleus with more than two red signals and no less than two green signals. A total of hundred cells with complete signals were counted and was defined positive when six presented cells were increased in hTERC copies (this threshold was determined after reviewing normal cervical cells from 20 patients). Positive represented increase in hTERC amplification.

***Statistical analysis***

Statistical analysis was performed with SPSS version 13.0 for Windows. The HPV L1 capsid protein expression and hTERC level were compared with chi square test and Spearman’s rank test. The sensitivity, specificity, positive predictive value and negative predictive value and diagnostic accuracy of HPV L1 capsid protein expression in cervical cells in prediction of CIN2/3 cervical lesions were determined. Receiver operating characteristic curve (ROC) was delineated and the area under curve (AUC) was calculated (A_Z_) and compared with chi square test and Z test. A value of P<0.05 was considered statistically significant. 

## Results

***Detection of HPV L1 capsid protein expression***

Among 309 patients, 142 were positive for HPV L1 capsid protein with the positive rate of 46.0%. With the increase of histological grade, the HPV L1 capsid protein expression had a decreasing tendency (χ^2^=24.84, *P*<0.001). In patients with CIN1, the HPV L1 capsid protein expression was markedly higher than that in patients with CIN2/3 (χ^2^=39.01, *P*<0.001) or SCC (χ^2^=38.40, *P*<0.001). The HPV L1 capsid protein expression was negatively related to the grade of cervical lesions (*r*_s_=-0.272, *P*<0.001), (Table 1). 

***Detection of*** ***hTERC gene***

Among 309 patients, 85 were positive for hTERC with a positive rate of 27.5%. With the increase in histological grade, the hTERC level had an increasing tendency (χ^2^=115.66,*P*<0.001). In patients with CIN1, the hTERC level was markedly lower than that in patients with CIN2/3(χ^2^= 69.06, *P*<0.001) or SCC (χ^2^=109.05, *P*<0.001). The hTERC level was positively related to the grade of cervical lesions (*r*_s_=0.605, *P*<0.001) (Table 1). 

***Detection of HPV L1 capsid protein and/or*** ***hTERC in diagnosis of CIN grade 2 or higher***

When diagnosis was done with findings in hTERC detection, the specificity, positive predictive value and accuracy were relatively high but the sensitivity was low (*P*<0.001). When diagnosis was done with findings in detection of HPV L1 capsid protein and hTERC, the negative predictive value was relatively high (*P*<0.05). When diagnosis was done with findings in detection of HPV L1 capsid protein, hTERC and HPV L1 capsid protein+ hTERC, the AUC was 0.704, 0.782 and 0.714, respectively, and they could be used to diagnose the CIN grade 2 or higher (*P*<0.001). However, z test confirmed that there was no difference in the screening of cervical cancer (*P*>0.05) (Table 2).

**Table 1:** HPV L1 capsid protein expression and hTERC gene in cervical cells

Pathology	Case	HPV L1 capsid protein		hTERC	
		Positive case	Positive Rate (%)	Positive case	Positive Rate (%)
NILM	33	9	27.3	2	6.1
CIN1	168	112	66.7	13	7.7
CIN2/3	84	21	25.0^a^	46	54.8^a^
SCC	24	0	0.0^a^	24	100.0^a^

^a^ *P*<0.001 vs CIN1 group

**Table 2:** Detection of HPV L1 capsid protein and hTERC in diagnosis of CIN grade 2 or higher

	Sensitivity (%)	Specificity (%)	PPV (%)	NPV (%)	Accuracy (%)	A_Z_
L1	80.6**	60.2**	52.1**	85.2	67.3**	0.704
hTERC	64.8	92.5	82.4	83.0	82.8	0.782
L1+ hTERC	89.8**	55.7**	52.2**	91.1*	67.7**	0.714

*P<0.05, **P<0.001 vs hTERC

***Correlation of cervical lesions with*** ***HPV L1 capsid protein expression and*** ***hTERC***

The HPV L1 capsid protein expression and hTERC had four combining forms: L1(+)/hTERC(+), L1(+)/hTERC(-), L1(-)/hTERC(+) and L1 (-)/hTERC (-). In patients with CIN1, the proportion of patients with L1(-)/hTERC(+) was lower than that in patients with CIN2/3 (χ^2^=14.628, *P*<0.001) or SCC (χ^2^=159.427, *P*<0.001). The L1(-)/hTERC(+) was positively related to the grade of cervical lesions (*P*<0.001), but L1(+)/hTERC(-) and L1(-)/hTERC(-) were negatively associated with the grade of cervical lesions (*P*<0.001). In addition, the constituent ratio of different forms was related to the grade of cervical lesions. The constituent ratio of L1(-)/hTERC (-)→L1(+)/hTERC (-)→L1(+)/hTERC(+)→L1 (-)/hTERC(+) had an increasing tendency with the increase in the grade of cervical lesions (χ^2^=189.84,*P*<0.001)(Table 3).

## Discussion 

The incidence of cervical cancer is increasing gradually in the developing countries, and early examination and early diagnosis of cervical cancer and precancerous lesions play an important role in clinical practice (15-19). Increasing attention has been paid to the role of HPV L1 capsid protein in the early diagnosis of cervical cancer (20-23). In the present study, the positive rate of HPV L1 capsid protein decreased with the increase in histological grade. In CIN1 patients, the HPV L1 capsid protein expression was higher than that in patients with CIN2/3 or SCC. These findings suggest that the expression of HPV L1 capsid protein is lost in CIN2/3 and SCC. HPV L1 is the major capsid protein of HPV (9, 24-26) and is expressed in early replication. When the HPV DNA is integrated into host DNA, the expression of HPV L1 capsid protein is lost in proliferative cells, and HPV L1 capsid protein is also a main target antigen inducing immune response following HPV infection (27-32). Our results showed that HPV L1 expression was negatively related to the grade of cervical lesions, which indirectly confirmed that the deficiency of HPV L1 was attributed to the progression of cervical lesions. McMurry *et al* (33) and Hagensee *et al* (34) explained that the loss of HPV L1 expression in early transformation phase could lead to an ineffective stimulation of immune response. Thus, the loss of HPV L1 expression may compromise the cellular immunity inducing the progression of cervical lesions. Thus, HPV L1 can be used as a biomarker in early examination and prediction of cervical lesions. hTERC locates at 3q26 chromosome region and encodes hTR which is a template in the synthesis of telomeric repeat and a core component in maintenance of telomerase activity. In the progression of cervical cells from atypical dysplasia to cervical cancer, the amplification of hTERC is almost inevitable and has been a promising candidate gene of atypical canceration of cervical cells (35). Our findings indicated the hTERC was positively related to the grade of cervical lesions, and the hTERC level increases with the increase in histological grade of cervical lesions. This also suggests that hTERC is a tumor marker in the malignant transformation of cervical intraepithelial neoplasia cells. Thus, hTERC can be used as a biomarker for the early diagnosis and the prediction of cervical lesions. 

**Table 3:** Different combining forms of HPV L1 and hTERC in different cervical lesions (%)

Pathology	L1-/hTERC-	L1+/hTERC-	L1+/hTERC+	L1-/hTERC+
NILM	22(66.7)	9(27.3)	0(0.0)	2(6.0)
CIN1	52(30.9)	103(61.3)	10(6.0)	3(1.8)
CIN2/3	27(32.1)	11(13.1)	10(11.9)	36(42.9^a^)
SCC	0(0.0^a^)	0(0.0^a^)	0(0.0)	24(100.0^ a^)
Total	101(32.7)	123(39.8)	20(6.5)	65(21.0)
x^2^	29.213	75.468	8.114	156.106
P	<0.001	<0.001	<0.05	<0.001
*r*_s_	-0.222	-0.339	0.083	0.612
*P*	<0.001	<0.001	>0.05	<0.001

^a^ *P*<0.001 vs CIN1

In the present study, histological findings served as gold standard and CIN grade two or higher served as positive and CIN grade one or lower as negative. When the diagnosis was performed with findings in hTERC detection alone, the specificity, positive predictive value and accuracy were relatively high, and detection of both HPV L1 capsid protein and hTERC had relatively high negative predictive value. Thus, the diagnostic value of detection of HPV L1 capsid protein and/or hTERC was compared with AUC of ROC. Results showed that the detection of HPV L1 capsid protein and/or hTERC could be used in the diagnosis of CIN gradetwo or higher, but the detection of both HPV L1 capsid protein and hTERC did not further increase the accuracy. This may be attributed to the negative for HPV L1 protein which reflects two statuses of viral DNA: viral DNA was integrated into host DNA resulting in malignant transformation; latent infection or absence of viral replication. This may also explain why the specificity and accuracy of HPV L1 capsid protein detection were lower than those of hTERC detection alone. 

In addition, our results also revealed that L1 (-)/hTERC(+) was positively related closely to the grade of cervical lesions (*r*_s_=0.612,*P*<0.001). In CIN1 patients, the proportion of L1(-)/hTERC(+) patients was lower than that in patients with CIN2/3 (42.9%) or SCC (100%), and this was the unique form in SCC patients. This indicates the loss of HPV L1 expression, and hTERC amplification plays an important role in the progression of cervical lesions. L1(-)/hTERC(+) also demonstrates that the HPV DNA has been integrated into host genome, and loss of HPV L1 expression and hTERC amplification may predict a high risk for cervical cancer. L1(+)/hTERC(+) was only found in CIN patients but not in SCC patients and those with normal cervical cells or chronic cervicitis. L1(+)/hTERC(+) means the presence of hTERC amplification, replication of HBP DNA, synthesis of HPV L1 and assembly of virosome. This form predicts high infectivity but can not be used to differentiate low grade from high grade. Thus, under the condition of L1(+)/hTERC(+), the grade of cervical lesions are uncertain. L1(+)/hTERC(-) was found in patients with normal cervical cells or chronic cervicitis or CIN1 or CIN2/3 but not in those with SCC. This implies that the HPV replication can induce cellular immunity but not hTERC amplification. L1(+)/hTERC(-) was negatively related to the grade of cervical lesions (r_s_=-0.339, *P*<0.001), and thus patients with L1(+)/hTERC(-) are regarded as having low risk for cervical cancer. L1(-)/hTERC(-) was not found in SCC patients. L1(-)/hTERC(-) means latent HPV infection, no or low HPV replication and no hTERC amplification. L1 (-)/hTERC(-) was negatively associated with the grade of cervical lesions (r_s_=-0.222, *P*<0.001) and patients with L1(-)/hTERC(-) can be regarded as having low risk. The L1/hTERC expression was in the following order with the increase in grade of cervical lesions: L1(-)/hTERC(-), L1(+)/hTERC(-), L1(+)/hTERC(+) and L1(-)/hTERC(+), which also reflects the progression of cervical lesions. Thus, we speculate that patients with L1(-)/hTERC(+) and CIN1 or lower should be closely monitored and cytological examination and biopsy may be regularly performed. For patients with L1(-)/hTERC(-) or L1(+)/hTERC (-) and CIN grade 1 or lower, the interval of follow up can be prolonged. For patients with L1(-)/hTERC(-) or L1(+)/hTERC(-) and CIN grade 2/3, physicians should be cautious about over-diagnosis and over-treatment. The findings in the present study required to be further confirmed in further studies. 

## Conclusion

The expression of HPV L1 capsid protein reduced with the increase of the histological grade of cervical cells and was negatively related to the grade of cervical lesions. However, the expression of hTERC increased with the increase of the histological grade and positively associated with the grade of cervical lesions. HPV L1 capsid protein and hTERC gene may serve as markers for the early diagnosis and prediction of cervical lesions. The increase in L1/hTERC ratio reflects the progression of cervical lesions to a certain extent.
